# An Update of Salivary Biomarkers for the Diagnosis of Alzheimer’s Disease

**DOI:** 10.3390/ijms26052059

**Published:** 2025-02-26

**Authors:** Haiying Guo, Ruihuan Yang, Weigao Cheng, Qiwen Li, Minquan Du

**Affiliations:** State Key Laboratory of Oral & Maxillofacial Reconstruction and Regeneration, Key Laboratory of Oral Biomedicine Ministry of Education, Hubei Key Laboratory of Stomatology, School & Hospital of Stomatology, Wuhan University, Wuhan 430079, China; haiyingguo@whu.edu.cn (H.G.); yangruihuan@whu.edu.cn (R.Y.); chengweigao@whu.edu.cn (W.C.); liqiwen@whu.edu.cn (Q.L.)

**Keywords:** salivary biomarkers, Alzheimer’s disease, salivary Aβ, salivary tau, salivary omics

## Abstract

Alzheimer’s disease (AD) is characterized by progressive cognition and behavior impairments. Diagnosing AD early is important for clinicians to slow down AD progression and preserve brain function. Biomarkers such as tau protein and amyloid-β peptide (Aβ) are used to aid diagnosis as clinical diagnosis often lags. Additionally, biomarkers can be used to monitor AD status and evaluate AD treatment. Clinicians detect these AD biomarkers in the brain using positron emission tomography/computed tomography or in the cerebrospinal fluid using a lumbar puncture. However, these methods are expensive and invasive. In contrast, saliva collection is simple, inexpensive, non-invasive, stress-free, and repeatable. Moreover, damage to the brain parenchyma can impact the oral cavity and some pathogenic molecules could travel back and forth from the brain to the mouth. This has prompted researchers to explore biomarkers in the saliva. Therefore, this study provides an overview of the main finding of salivary biomarkers for AD diagnosis. Based on these available studies, Aβ, tau, cholinesterase enzyme activity, lactoferrin, melatonin, cortisol, proteomics, metabolomics, exosomes, and the microbiome were changed in AD patients’ saliva when compared to controls. However, well-designed studies are essential to confirm the reliability and validity of these biomarkers in diagnosing and monitoring AD.

## 1. Introduction

Alzheimer’s disease (AD) is a progressive neurodegenerative disease and gradually deteriorates patients’ memory, cognition, and ability to perform daily activities [1]. According to the World Health Organization (WHO), AD is the seventh leading cause of death among all diseases worldwide [2]. AD is characterized by the accumulation of extracellular amyloid-β (Aβ) peptide plaques and intracellular neurofibrillary tangles [3,4]. AD progresses with age along a continuum with three major phases: preclinical AD with only pathophysiological changes, mild cognitive impairment due to AD (aMCI), and clinically apparent dementia due to AD (AD-dementia) [5,6]. Currently, the diagnosis of AD relies largely on clinical symptoms. However, the symptoms at the early stages of AD are latent and insidious and are frequently overlooked by both patients and doctors. This leads to a large number of AD patients being diagnosed late or not at all. Worse still, few effective drugs are available to reverse the progression of AD [7]. Studies show that the early detection of AD could preserve brain function, improve people’s quality of life, and help reduce the burden on the aging society worldwide [6,7,8]. Thus, finding methods for detecting AD early and monitoring the progression of AD is important.

A growing number of studies have identified biomarkers, reflecting the pathophysiological changes of AD in the brain and the cerebrospinal fluid (CSF), to detect and monitor AD [9,10]. Common biomarkers are Aβ peptide, phosphorylated tau (p-tau) protein, and total tau (t-tau) protein in the brain and CSF [11]. The detection methods for biomarkers in the CSF are cheap, whilst the method of extracting them, such as lumbar puncture, is invasive and difficult to perform regularly [12,13]. These biomarkers in the brain can be detected using positron emission tomography/computed tomography but this method is expensive [14]. Therefore, researchers are exploring non-invasive and affordable methods to detect and monitor AD.

**Figure 1:**
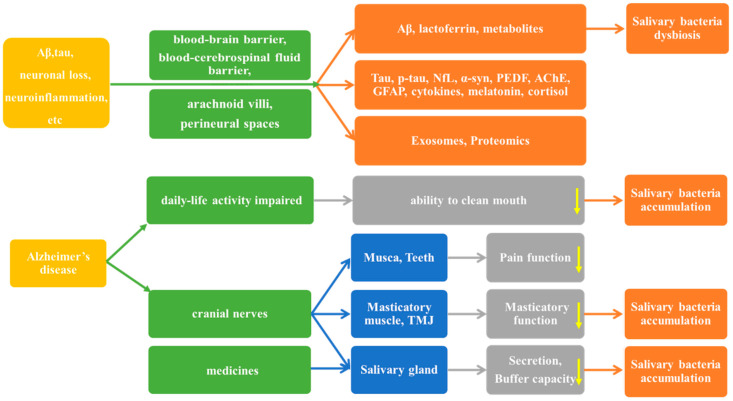
The pathway of Alzheimer’s disease impacts the saliva. The yellow arrow represents a decline in function.

It is well-known that saliva collection is straightforward and non-invasive. The pathogenic molecules associated with AD can travel back and forth from the brain to the saliva via the blood–brain barrier, the blood–cerebrospinal fluid barrier, arachnoid villi, and perineural spaces [15,16,17]. For instance, brain-derived Aβ peptides are transported into the periphery via the blood–brain barrier and vice versa [18]. It is, therefore, possible to use salivary biomarkers to detect and monitor AD. At the same time, some pathogenic molecules, including Aβ and lactoferrin, are also antimicrobial peptides, and they can affect the salivary microbiome [19,20]. In addition, secretions from the salivary glands and mastication, controlled by the cranial nerves, decreased in people with AD, further leading to the disturbance of the salivary microbiome [21,22,23,24,25,26]. People with AD have difficulty in cleaning the oral cavity and this can also result in a bacteria explosion [27]. Moreover, some medicines taken by people with AD reduce salivary flow rate and buffering capacity, such as anticholinesterases and memantine [28]. Thus, other than the salivary molecules, monitoring the changes in the salivary microbiome is also a method of detecting AD (details shown in Figure 1).

The aim of this study is to provide a concise review of potential biomarkers, including the salivary microbiome, for the detection and monitoring of AD, and to find salivary biomarkers that could be used to detect AD in the early stages.

## 2. Results

A total of 3868 articles were obtained by searching online databases and a further 22 articles were identified through cross-referencing. After removing duplicate papers and screening abstracts, 45 relevant papers remained. Among them, 41 papers studied the potential utility of salivary molecules to distinguish AD from controls and four papers examined the salivary microbiome.

There were two categories of AD biomarkers in saliva. One category was the direct biomarkers of the brain damage that occurs in AD, including Aβ, tau, cholinesterase enzyme activity, biomarkers of neurodegeneration or neuronal injury, and neuroinflammation cytokines. The other included the indirect biomarkers of AD, such as lactoferrin, melatonin, cortisol, proteomics, metabolomics, exosomes, and the microbiome.

### 2.1. Direct Biomarkers of the Pathological Changes of AD

#### 2.1.1. Aβ

Aβ is produced by the cleavage of the amyloid precursor protein (APP) by α, β, and γ secretases [29]. The cleavage of APP by α and γ secretases produces a soluble APP (sAPPα) outside the cell membrane, a small p3 fragment in the extracellular space, and an APP intracellular domain [30]. The cleavage of APP by β and γ produces a large soluble APP derivative (sAPPβ), the Aβ and APP intracellular domain [31]. As the γ secretase cleaves APP at various sites, Aβ can have different chain lengths, ranging from 38 to 43 residues [32]. Among them, Aβ40 and Aβ42 are the common types involved in AD [33]. Aβ42 is the main component of amyloid plaques in AD [32]. Aβ42 levels and the Aβ42/Aβ40 ratio in the CSF are common clinical indexes used to diagnose AD [34]. Notably, brain-derived Aβ peptides are transported into the periphery via the blood–brain barrier and vice versa [18]. Saliva reflects the changes in the CSF [35]. Thus, it is possible to use the changes in peripheral Aβ to screen for or detect AD.

The results of studies on salivary Aβ42 have been diverse. Sabbagh et al. and Lee et al. used an enzyme-linked immunosorbent assay (ELISA) to analyze its changes and found that the salivary Aβ42 levels in the AD group were over twice those of the control group [36,37]. Similar to their findings, seven other studies also reported an increase in Aβ42 levels in AD patients relative to controls [38,39,40,41,42,43,44]. Moreover, salivary Aβ42 levels were lower during the mild cognitive impairment stage than in the AD stage [41,44]. Specifically, in a study, the salivary Aβ42 level in the high-level control group (composed of people with a family history of AD), was nearly 1.8 times higher than that of the low-level controls (37.96 ± 8.13 pg/mL versus 21.26 ± 1.73 pg/mL) [40]. Moreover, salivary Aβ42 levels were positively correlated with the levels of total tau (t-tau) and phosphorylated tau (p-tau) in the CSF [38]. These results suggested an increase in salivary Aβ42 levels as AD progresses. However, there are conflicting results. Tvarijonaviciute et al. used an immunology multiplex assay (IMA) and detected a lower level of salivary Aβ42 in the AD group compared to the control group [45]. Additionally, three studies failed to detect Aβ42 in the saliva of AD patients [46,47,48] (details shown in Table 1).

For salivary Aβ40, the situation is also inconsistent. Kim et al., using a magnetic nanoparticle immunoassay (MNI), found that salivary Aβ40 levels were higher in the AD group than in the control group [41]. Conversely, two studies that selected ELISAs to detect salivary Aβ40, found no difference between the AD patients and the controls [39,43]. Meanwhile, Marksteiner et al. did not detect Aβ40 in saliva [48] (details shown in Table 1 and Appendix A Table A1).

#### 2.1.2. Tau Protein

Tau is a microtubule-associated protein and plays a key role in maintaining the microarchitecture of neuronal axons [49]. The biological activity of tau is regulated by its phosphorylation [50]. Abnormal phosphorylation causes tau to lose its affinity for microtubules, leading it to aggregate and form intracellular neurofibrillary tangles [49]. The degree of p-tau is related to the degree of neuronal damage in the brain and the progression of AD [34]. Therefore, the level of p-tau and the ratio of p-tau/t-tau can be used for the early detection of AD and to predict the rate of cognitive decline in AD subjects [51]. Interestingly, extracellular soluble tau in the brain can be transported into peripheral tissues by arachnoid villi, the blood–cerebrospinal fluid barrier, and perineural spaces [52]. The saliva reflects the changes in the CSF [35]. Hence, salivary tau levels can serve as a reflection of tau changes in the brain.

Six studies examined salivary p-tau levels. Among them, four studies used ELISAs to detect salivary p-tau and obtained different results. Specifically, Katsipis et al. and Sabaei et al. found that salivary p-tau levels were higher in the AD group than in the control group [42,44]. Meanwhile, Lau et al. and Cui et al. did not find differences in salivary p-tau between AD patients and controls [39,47]. In addition, one study, using mass spectrometry, detected higher salivary p-tau levels in the AD group when compared with the control group [46]. Meanwhile, another study found no difference in salivary p-tau across the two groups [45]. As for salivary t-tau, there were also six studies that analyzed the changes between the two groups. These six studies used different methods to detect salivary t-tau. Five studies found that there was no difference in salivary t-tau between the two groups [39,45,46,47,53]. Only one study found that salivary t-tau, detected by lumipulse assays, was lower in the AD group than the control group, and the decrease in salivary t-tau levels was markedly pronounced in female AD patients but not in male AD patients [48]. Moreover, contradictory findings emerged regarding alterations in the p-tau/t-tau ratio between groups across studies [39,46,54]. Notably, one study reported no difference in the ratio between the two groups [39]. Meanwhile, results from mass spectrometry and Western blots both showed an elevation of this ratio in AD patients [46,54]. Paradoxically, despite the observed ratio increase in AD, subsequent analyses revealed no significant correlations between the p-tau/t-tau ratio and key disease indicators, including CSF tau levels, hippocampal atrophy, or neuropsychological test performance [54] (details shown in Table 2 and Appendix A Table A2).

#### 2.1.3. The Salivary Biomarkers of Neurodegeneration or Neuronal Injury

Neurodegeneration and neuronal injury are key pathophysiological features of AD. As a result of the neuronal damage in the central nervous system (CNS), neurofilament light chains (NfL), the most important subunit for axonal radial growth, are released into the blood and CSF [55]. Studies found that both CSF NfL and plasma NfL levels were elevated in AD patients [56,57]. On the other hand, Gleerup et al. did not find a difference in salivary NfL between AD patients and controls [58]. α-synuclein (α-syn) is an intrinsically disordered protein and is involved in the pathology of Aβ and tau [59]. CSF α-syn in the AD group was increased when compared with the control group [60]. As for salivary α-syn, it was decreased in the AD group [42]. Pigment epithelium-derived factor (PEDF) is a unique neurotrophic protein that negatively regulates Aβ42 [61]. Serum PEDF was decreased in AD patients when compared to the controls [61]. Tvarijonaviciute et al. found that there was no difference in salivary PEDF between the two groups [45] (details shown in Table 3 and Appendix A Table A3).

#### 2.1.4. Acetylcholinesterase (AChE) Activity in Saliva

AChE is produced by cholinergic neurons. It is expressed both in the brain and saliva. The key neurochemical disorder in the brain of AD patients is a marked decrease in AChE activity [62]. Some researchers intended to use the changes in salivary AChE activity to diagnose AD. Sayer et al. discovered that salivary AChE activity decreased to a greater extent in the AD group compared to the control group [63]. Meanwhile, Ahmadi et al. found that salivary AChE activity increased in individuals with AD compared to controls [64]. On the other hand, Bakhtiari et al. and Boston et al. found no differences in salivary AChE activity between the AD group and the control group [65,66] (details shown in Table 4 and Appendix A Table A4).

#### 2.1.5. Neuroinflammation Markers in Saliva

Apart from amyloid and tau pathologies, overwhelming evidence shows that neuroinflammation plays a prominent role in the progression of AD [67]. Microglia and astrocytes are the primary immune cells involved in neuroinflammation. Upon activation, glial cells secrete a variety of inflammatory cytokines, such as interleukin(IL)-1, IL-6, tumor necrosis factor (TNF), cyclooxygenase-2 (COX-2), caspase-8, matrix metalloproteinase 9 (MMP-9), macrophage inflammatory protein-4 (MIP-4), and so on [68].

Studies found that salivary IL-1, IL-6, interleukin-1 receptor antagonist (IL-1RN), and TNF-α decreased to a greater extent in the AD group than in the control group, while complement C4 (CC4), COX-2, caspase-8, IL-1β, and MMP-9 showed opposite results [21,44,45,69]. Meanwhile, no difference was found between the two groups for MIP-4 and C-reactive protein (CRP) [45] (details shown in Table 5). As stated above, astrocytes are responsible for reactive hyperplasia in AD. Glial fibrillary acidic protein (GFAP) is the structural protein of astrocytes. Thus, GFAP levels are elevated as a result [70]. Unexpectedly, Katsipis et al. found that salivary GFAP levels were decreased in AD patients, which contradicted the findings of GFAP in the brain and blood [44]. Moreover, GFAP levels decreased as AD progressed [44] (details shown in Table 5 and Appendix A Table A5).

### 2.2. The Indirect Biomarkers of AD in Saliva

#### 2.2.1. Lactoferrin

Lactoferrin, an iron-binding glycoprotein, is usually present in the milk, saliva, seminal fluid, mucosal surfaces, and secondary granules of neutrophils [71,72]. Its common function is to promote iron absorption in the human body. Lactoferrin also plays a crucial role in defending against invading pathogens through its antimicrobial properties [73]. Moreover, lactoferrin present in the human brain has several neuroprotective benefits [74,75]. For example, the anti-inflammatory and antioxidant functions of lactoferrin repair neurons and protect the integrity of the brain [76,77]. Furthermore, lactoferrin participates in the pathology of AD. That is, lactoferrin is elevated in the brain of AD patients, and it is deposited in Aβ peptide plaques and intracellular neurofibrillary tangles [77,78,79]. It is worth noting that lactoferrin could be rapidly transported across the blood–brain barrier, which marks the demarcation between peripheral and central systems [79]. Thus, we can try using lactoferrin for the early detection of AD and for monitoring its progression.

Carro’s team and Zalewska’s team both found that salivary lactoferrin levels decreased in the AD group compared with the control group [21,80,81]. Carro’s team also found that salivary lactoferrin levels were decreased in patients with late-onset AD compared to early-onset AD, while the impact of age on lactoferrin was lost in control groups [82]. In addition, salivary lactoferrin levels were positively correlated with CSF Aβ42 and negatively associated with CSF t-tau [80]. Moreover, salivary lactoferrin levels were negatively associated with cerebral amyloidosis in the brain [81]. Both these results suggested that salivary lactoferrin could be used to detect AD. Meanwhile, another study did not discover a difference in salivary lactoferrin among healthy controls and AD patients [83]. Furthermore, there were no relationships between lactoferrin levels and CSF tau, CSF p-tau, and CSF Aβ42 [83] (details shown in Table 6 and Appendix A Table A6).

#### 2.2.2. Salivary Melatonin

Circadian dysfunction, a key symptom of AD, is found even in the mild and moderate stages of AD [84]. Melatonin, a neurohormone, regulates the circadian rhythms. A decrease in melatonin is detected in the early stages of AD, even before the onset of clinical symptoms [85]. Moreover, melatonin could be transported into the peripheral tissue [86]. Given this, Manni et al. used ELISAs to detect salivary melatonin and found that melatonin secretion onset by dim light decreased in AD patients compared to controls [87]. Meanwhile, no difference in melatonin level, as detected by radioimmunoassays, was found between patients with mild AD and controls in the study conducted by Weissova et al. [88] (details shown in Table 7).

#### 2.2.3. Salivary Cortisol

Studies show that cortisol levels are evaluated in AD patients, and high cortisol contributes to AD via exacerbating Aβ and tau pathology [89,90]. Three studies aimed to examine changes in salivary cortisol in AD patients. Giubilei et al. found that salivary cortisol levels were higher in AD patients than in controls, being negatively correlated with mini mental state examination (MMSE) scores and positively correlated with cerebral atrophy indexes [91]. Meanwhile, Pena-Bautista et al. and James et al. found no difference in salivary cortisol between the AD group and the control group [92,93] (details shown in Table 8 and Appendix A Table A7).

#### 2.2.4. Oxidative Stress Markers in Saliva

It has been shown that mitochondrial oxidative stress plays a key role in the pathogenesis of AD [94]. Importantly, concentrations of oxidative proteins are increased in both the CNS and peripheral nervous system. Two independent studies quantitatively analyzed oxidative stress markers in both AD patients and healthy controls. The results demonstrated a distinct redox imbalance in AD pathophysiology: (1) some glutathione peroxidase activity was reduced in the AD group; (2) some oxidative damage biomarkers showed marked elevations compared to controls; while (3) some redox-regulatory proteins remained comparable between groups [21,45]. For example, there was a decrease in the activity of superoxide dismutase and catalase in the AD group with respect to the control group (details shown in Table 9 and Appendix A Table A8) [21]. Moreover, a positive correlation between peroxidase and the time elapsed from AD diagnosis was observed in this study [21].

#### 2.2.5. The Biomarkers of AD in Salivary Exosomes

Exosomes are vehicles for proteins, DNA, mRNA, miRNA, and other non-coding RNAs (ncRNAs) and are involved in Aβ-amyloid deposits, the formation of NFTs, neuroinflammation, and neuronal dysfunction in AD [95,96]. Exosomes in the brain can be released into saliva [97]. In addition, those in the cerebrospinal fluid can be released into the systemic circulation and subsequently into saliva [98]. One study found that the expression of oligomeric Aβ and phosphorylated tau in salivary exosomes in AD patients was higher than in healthy people, and the expression of the Aβ monomer was lower [99]. One study found that the miRNA-485-3p concentrations in salivary exosomes in the AD group were increased compared with the control group (details shown in Table 10 and Appendix A Table A9) [100].

#### 2.2.6. Salivary Proteomics

Apart from proteins from the salivary glands, there are other components in salivary proteomics. Notably, about 19.8% of salivary proteins are shared with the CSF [101]. In view of this, some studies used salivary proteomics to detect AD. One study used liquid chromatography/mass spectroscopy (LC-MS/MS) and Western blots to detect salivary proteins in both AD patients and healthy controls, and found that the protein level of salivary transthyretin decreased in the AD group compared to the controls [101]. One study found that some proteins in tyrosine metabolism, pyruvate metabolism, glycolysis, antigen processing and presentation, and leukocyte transendothelial migration were changed in the AD group compared to the control group [102]. For example, ALDH3 displayed decreased expression in the AD group [102]. One study found higher levels of some salivary proteins in the AD group than in the control group [103]. For example, cystatin B, which has antimicrobial activity, and thymosin β4, which has a neuroprotective role, were enriched in the AD group [103] (details shown in Table 11 and Appendix A Table A10). Moreover, they found that the abundance of the cystatin B interactome was different between the two groups [104]. Triosophosphate isomerase 1 exhibited lower levels in the AD group, and there was a higher level of mucin 7 in the AD group with respect to the control group [104].

#### 2.2.7. Salivary Metabolites

The saliva metabolome appears to be comparable to the CSF metabolomes in terms of chemical complexity and the number of compounds [105]. Several studies detected salivary metabolites to find some biomarkers to distinguish AD from controls. For instance, acyl-alkyl phosphatidylcholines in the saliva of AD patients decreased compared to controls [106]. The concentrations of acetone and propionate increased in AD groups [107]. Additionally, the concentrations of methylguanosine, histidinyl-phenylalanine, and choline-cytidine increased in AD patients, and the panel of three metabolites demonstrated excellent diagnostic accuracy in distinguishing AD from the control group [108]. One study found that sphinganine-1-phosphate, ornithine, and phenyllactic acid were upregulated in AD patients compared to the controls, and satisfactory performance was achieved with a sensitivity of 99.4%, and a specificity of 98.2% [109]. Another study used gas chromatograph-mass spectrometry to analyze salivary metabolites and found that succinate, fumarate, and L-lactate were downregulated in AD patients [102] (details shown in Table 12, and Appendix A Table A11 and Table A12).

#### 2.2.8. Potential AD Biomarkers in the Salivary Microbiome

As stated above, there are several pathways through which the salivary microbiome is impacted by AD. There were four studies that detected the salivary microbiome in both AD patients and controls. The characteristics varied among these studies. Three studies excluded individuals who had taken antibiotics and/or received dental treatments prior to saliva collection [111,112,113]. However, in the study conducted by Bathini et al., it was unclear whether individuals who had taken antibiotics and/or received dental treatments were included or excluded [114]. Only one study specified the minimum number of teeth [111]. Two studies used oral health examinations and found that there were no differences in the oral health of AD patients and controls [111,113]. Meanwhile, the other two studies did not report on oral status [112,114]. Three studies utilized V3-V4 16S rRNA sequencing on the Illumina platform [112,113,114] and one study used 16S rRNA full-length sequencing on the PacBio platform [111]. The taxonomic assignment strategies were different among these studies. Two studies used the operational taxonomic unit (OTU) [112,113], one study used amplicon sequence variants (ASVs) [111], and the taxonomic assignment strategy was unclear in the study conducted by Bathini et al. [114] (details shown in Table 13 and Table 14).

##### α and β Diversity of the Salivary Microbiome

α diversity analysis measures the diversity, evenness, and richness of the salivary microbiome between groups, and β diversity analysis tests the similarity or dissimilarity of the community of the salivary microbiome between groups. One study used 16S ribosomal RNA (16S rRNA) sequencing and found that the Chao 1 index, an estimator measuring species richness, was lower in AD patients than in healthy controls [112]. Meanwhile, Bathini et al. did not find a difference in the Shannon index between the two groups [114]. Another two studies did not report Chao1 and Shannon index results [111,113]. Guo et al. and Liu et al. found that the community composition of the salivary microbiome was similar between groups [111,112], while Fu et al. found a difference in the community composition of the salivary microbiome between the AD group and the control group [113] (details shown in Table 14).

##### Significant Bacteria of the Salivary Microbiome Between Groups

Though there were different results regarding the α and β diversity of the salivary microbiome between groups, all of the included studies found some significantly different bacteria. For instance, Fu et al. found a higher abundance of *Eubacterium infirmum*, *Prevotella buccae*, and *Selenomonas artemidis* in the AD group than in the control group [113]. Bathini et al. found that the relative abundance of *Filifactor villosus* decreased in the AD group compared to the control group, and it declined with the increasing severity of AD [114]. The relative abundance of *Veillonella parvula* increased in AD patients when compared with the controls in the study conducted by Guo et al., and it was positively associated with AD [111]. A study found that the relative abundance of *Moraxella*, *Leptotrichia*, and *Sphaerochaeta* in the AD group increased compared with the control group, whereas that of *Rothia* decreased [112]. Moreover, no bacteria were found to be associated with the severity of AD [112] (details shown in Table 14).

## 3. Discussion

As mentioned above, an increasing number of studies have paid attention to the use of salivary biomarkers in the diagnosis of or screening for AD. However, inconsistent changes and uncertainties regarding the specificity, sensitivity, and reliability of these salivary biomarkers limit their widespread use.

The main reason that explains the inconsistent changes in salivary biomarkers is the heterogeneity of the included subjects in the studies. This heterogeneity can be attributed to three main factors. Firstly, the diagnostic criteria of AD are different among studies, leading to the heterogeneity of included people. Some studies used the diagnostic criteria recommended by National Institute on Aging Alzheimer’s Association (NIA-AA) workgroups [36,38,42,54,69,80,82,83,87,88,93], while others diagnosed AD according to the National Institute on Neurological Communicative Disorders and Stroke and the Alzheimer’s Disease and Related Disorders Association guidelines (NINCDS–ADRDA) [46,66,91,102,106,109]. Notably, the clinical symptom, dementia, is required for an AD diagnosis according to the guidelines from NINCDS–ADRDA, while no clinical symptoms are required on the basis of recommendations from the NIA-AA [115]. The NIA-AA recommends that preclinical AD is diagnosed solely by detecting the levels of Aβ and tau in the CSF or in the brain. There is an overlap between preclinical AD and intact cognition with only clinical mental examinations [4,13], meaning that some preclinical AD will be mistaken for the controls in some studies. For example, the study conducted by Pena-Bautista et al. only recruited healthy people with negative biomarkers of AD (CSF Aβ and CSF tau) in the control group [93], while Giubilei et al. did not exclude people with preclinical AD from the control group [91]. The heterogeneity of the control group led to different results in salivary cortisol in these two studies. In addition, the severity of AD varied across the studies, and the concentrations of salivary biomarkers changed along with the severity of AD. For example, there was a positive correlation between salivary cortisol levels and AD severity [116]. Therefore, the different severity levels of AD likely contributed to the heterogeneity of subjects in studies and further impacted the consistency of salivary biomarkers. A study that included only mild dementia patients in the AD group did not find a difference in melatonin levels between the two groups [88], while the study that included people with moderate dementia in the AD group found a decrease in melatonin onset under dim light secretion in the AD group compared to the control group [87]. Meanwhile, the various severity levels of AD among the two studies were the reason why they obtained different results regarding salivary lactoferrin in AD patients [81,83]. The third reason is the different grouping methods for patients with AD. The study only recruited AD patients who did not respond to AChE-I therapy (non-responsive AD) and found that salivary AChE activity decreased to a greater extent in AD patients than in controls [63]. Meanwhile, there was no difference in salivary AChE activity between the AD group and the control group in the study that did not group AD patients on the basis of their response to AChE-I therapy [66].

In addition to the inconsistency, the specificity, sensitivity, and reliability of these salivary biomarkers were uncertain, limiting their widespread use. Some biomarkers are not specific to AD, and they require further validation to assess their specificity. For example, melatonin is the biomarker of circadian dysregulation [117]. Cortisol is the common biomarker of chronic stress [118]. α-syn is the biomarker of Parkinson’s disease [119]. Cytokine levels often increase during infectious diseases and are not reliable biomarkers. In addition, the minimal accumulation of these biomarkers in the brain, as reflected in the saliva, is unclear. Thus, we could not ensure the sensitivity of salivary biomarkers for the early detection of AD. Lastly, with a high false positive rate, certain salivary omics biomarkers (salivary proteomics, metabolomics, and the microbiome) require further validation in basic experiments to study how AD impacts them. This would allow us to ensure the reality of these biomarkers.

Except for the characteristics of some salivary biomarkers stated above, other shortcomings of the design of these included studies limit the wide use of salivary biomarkers to detect and monitor AD. Firstly, the lack of early stages in AD (preclinical AD and aMCI) and the limited sample size result in the included individuals not being representative of the target population, and the results could not be generalized to the population. Secondly, the oral status of the individuals is unclear, and oral diseases impact the levels of salivary biomarkers. For example, the concentrations of Aβ, lactoferrin, and IL-6, and the relative abundance of *Porphyromonas gingivalis* in the saliva of periodontitis patients, increased when compared with those of healthy controls [120,121,122]. Without accounting for oral health status, we cannot determine whether these salivary biomarkers are produced by AD or oral diseases. Thirdly, other confounders including age, saliva type, collection methods, measurement, medicines, and conservation products impact the concentrations of salivary biomarkers. As for salivary Aβ, its level increased with age and decreased with ibuprofen treatment [40,123]. Without the conservation product thioflavin S, the solute Aβ42 can easily self-aggregate, which reduces its concentration [37,47]. As for lactoferrin, its concentration in whole unstimulated saliva was low when compared to that of parotid saliva [121], and its concentration in stimulated saliva was lower than that of unstimulated saliva [124]. The concentration of cortisol was reduced in saliva collected by cotton compared to saliva collected by spitting [125]. In salivary omics studies, variations in sequencing platforms, assessment methods, and computational algorithms also contribute to discrepancies in biomarker levels across different studies.

In summary, there are differences in the molecules and bacteria of individuals with AD and those with intact cognition. However, utilizing these molecules as biomarkers for the early detection of AD is still a distant goal. In the future, salivary biomarkers can be used for the early diagnosis of AD if numerous studies meet the following criteria: a sufficient sample size, comprehensive subgroups of AD patients (including preclinical AD, aMCI, and AD-dementia), standardized collection and measurement methods, balanced oral health statuses across groups, and adequate control for confounders.

## 4. Materials and Methods

The following keywords, #1, oral OR dental OR saliva; #2, biomarker OR Aβ OR tau OR lactoferrin OR microbiome; and #3, Alzheimer’s disease, as well as the search strategy ((#1 AND #2) AND #3), were used to search the PubMed, Embase, Web of Science, and Cochrane library databases. The search was restricted to publications in English, with no publication year limit. The last search was performed on 18 July 2024. A researcher reviewed the titles, abstracts, and reference lists to select pertinent publications. Only original papers on the salivary biomarkers of AD were considered. Additionally, the references of included articles were also manually retrieved to maximize the number of included studies. Moreover, studies where the full text could not be assessed were excluded.

## Figures and Tables

**Table 1:** The changes in salivary Aβ in people living with AD.

Authors (Year)	Group: No.	Saliva Type	Collection Method	Sodium Azide	Thioflavin S	Assessment Method	Main Findings	
							Aβ42	Aβ40
Bermejo et al. (2010) [43]	AD: 29 Control: 56	Unstimulated whole saliva	Spit	Yes	Unreported	ELISA	AD > Control	No difference
Boschi et al. (2022) [38]	AD: 18 Control: 18	Unstimulated whole saliva	Spit	Yes	Yes	ELISA	AD > Control	NA
Cui et al. (2022) [39]	AD: 30 Control: 30	Unstimulated parotid saliva	Swab	Unreported	Unreported	ELISA	AD > Control	No difference
Katsipis et al. (2021) [44]	AD: 20 Control: 20	Unstimulated whole saliva	Spit	Unreported	Unreported	ELISA	AD > Control	NA
Kim et al. (2014) [41]	AD: 28 Control: 17	Unstimulated whole saliva	Spit	Yes	Unreported	MNI	AD > Control	AD > Control
Lee et al. (2017) [37]	AD: 7 Control: 26	Unstimulated whole saliva	Spit	Yes	Yes	ELISA	AD > Control	NA
McGeer et al. (2020) [40]	AD: 30 Control: 237	Unreported	Unreported	Yes	Yes	ELISA	AD > Control	NA
Sabaei et al. (2023) [42]	AD: 24 Control: 22	Unstimulated whole saliva	Cotton	Unreported	Unreported	ELISA	AD > Control	NA
Sabbagh et al. (2018) [36]	AD: 15 Control: 7	Unstimulated whole saliva	Spit	Yes	Yes	ELISA	AD > Control	NA
Tvarijonaviciute et al. (2020) [45]	AD: 69 Control: 83	Unstimulated whole saliva	Spit	Unreported	Unreported	IMA	AD < Control	NA
Lau et al. (2015) [47]	AD: 20 Control: 20	Unstimulated whole saliva	Spit	Unreported	Unreported	ELISA	Not detected	NA
Marksteiner et al. (2022) [48]	AD: 44 Control: 27	Unstimulated whole saliva	Spit	Unreported	Unreported	Lumipulse Assay	Not detected	Not detected
Shi et al. (2011) [46]	AD: 21 Control: 38	Unstimulated whole saliva	Cotton	Unreported	Unreported	Mass spectrometry	Not detected	NA

Abbreviations: AD, Alzheimer’s Disease; ELISA, enzyme-linked immunosorbent assay; IMA, immunology multiplex assay; MNI, magnetic nanoparticle immunoassay; NA, not applicable.

**Table 2:** The changes in salivary tau in people living with AD.

Authors (Year)	Group: No.	Saliva Type	Collection Method	Inhibitor	Assessment Method	Main Findings		
						p-tau	t-tau	p-tau/t-tau
Cui et al. (2022) [39]	AD: 30 Control: 30	Unstimulated parotid saliva	Swab	Unreported	ELISA	No difference	No difference	No difference
Katsipis et al. (2021) [44]	AD: 20 Control: 20	Unstimulated whole saliva	Spit	Unreported	ELISA	AD > Control	NA	NA
Marksteiner et al. (2022) [48]	AD: 44 Control: 27	Unstimulated whole saliva	Spit	Unreported	Lumipulse assay	NA	AD < Control	NA
Pekeles et al. (2019) [54]	AD: 46 Control: 47	Unstimulated whole saliva	Spit	Yes	Western Blot	NA	NA	AD > Control
Sabaei et al. (2023) [42]	AD: 24 Control: 22	Unstimulated whole saliva	Cotton	Unreported	ELISA	AD > Control	NA	NA
Shi et al. (2011) [46]	AD: 21 Control: 38	Unstimulated whole saliva	Cotton	Unreported	Mass spectrometry	No difference	No difference	AD > Control
Ashton et al. (2018) [53]	AD: 53 Control: 160	Unstimulated whole saliva	Spit	Unreported	Simoa	NA	No difference	NA
Lau et al. (2015) [47]	AD: 20 Control: 20	Unstimulated whole saliva	Spit	Yes	ELISA	No difference	No difference	NA
Tvarijonaviciute et al. (2020) [45]	AD: 69 Control: 83	Unstimulated whole saliva	Spit	Unreported	IMA	No difference	No difference	NA

Abbreviations: AD, Alzheimer’s Disease; ELISA, enzyme-linked immunosorbent assay; IMA, immunology multiplex assay; NA, not applicable; Simoa, single molecule array.

**Table 3:** The salivary biomarkers of neurodegeneration or neuronal injury.

Authors (Year)	Group: No.	Saliva Type	Collection Method	No Smoking	Assessment Method	Main Findings
Gleerup et al. (2021) [58]	AD: 49 Control: 17	Unstimulated whole saliva	Spit	Yes	Simoa	NfL: No difference
Sabaei et al. (2023) [42]	AD: 24 Control: 22	Unstimulated whole saliva	Cotton	Unreported	ELISA	α-syn: AD < Control
Tvarijonaviciute et al. (2020) [45]	AD: 69 Control: 83	Unstimulated whole saliva	Spit	Yes	Immunoassays	PEDF: No difference

Abbreviations: AD, Alzheimer’s Disease; ELISA, enzyme-linked immunosorbent assay; NfL, neurofilament light chain; α-syn, alpha-synuclein; PEDF, pigment epithelium derived protein; Simoa, single molecule array.

**Table 4:** Salivary acetylcholinesterase activity.

Authors (Year)	Group: No.	Saliva Type	Collection Method	Medicine Using	Assessment Method	Main Findings
Ahmadi et al. (2019) [64]	AD: 30 Control: 30	Unstimulated whole saliva	Spit	Unreported	Ellman colorimetric	AD > Control
Sayer et al. (2004) [63]	AD: 14 Control: 11	Unstimulated whole saliva	Spit	AchE-I	Ellman colorimetric	AD < Control
Bakhtiari et al. (2017) [65]	AD: 15 Control: 15	Unstimulated whole saliva	Spit	Memantine	Ellman colorimetric	No difference
Boston et al. (2008) [66]	AD: 15 Control: 13	Unstimulated whole saliva	Spit	Anticholinergics	Ellman colorimetric	No difference

Abbreviations: AchE, acetylcholinesterase; AchE-I, acetylcholinesterase inhibitors; AD, Alzheimer’s Disease.

**Table 5:** Salivary cytokines.

Authors (Year)	Group: No.	Saliva Type	Collection Method	Protein Stabilizing	Assessment Method	Main Findings
Katsipis et al. (2021) [44]	AD: 20 Control: 20	Unstimulated whole saliva	Spit	Unreported	ELISA	AD < Control: IL-1, IL-6, TNF-α, GFAP AD > Control: COX-2, Caspase-8
McNicholas et al. (2022) [69]	AD: 16 Control: 29	Unreported	Absorbent pad	Yes	ELISA	AD < Control: IL-1RN AD > Control: MMP-9
Tvarijonaviciute et al. (2020) [45]	AD: 69 Control: 83	Unstimulated whole saliva	Spit	Unreported	Immunoassays	AD > Control: CC4 No difference: MIP-4, CRP
Zalewska et al. (2021) [21]	AD: 25 Control: 25	Stimulated whole saliva	Suction	Unreported	ELISA	AD > Control: IL-1β

Abbreviations: AD, Alzheimer’s Disease; CC4, complement C4; COX-2, cyclooxygenase-2; CRP, C-reactive protein; ELISA, enzyme-linked immunosorbent assay; GFAP, glial fibrillary acidic protein; IL, interleukin; IL-1RN, interleukin-1 receptor antagonist; MMP-9, matrix metalloproteinase 9; MIP-4, macrophage inflammatory protein-4; TNF-α, tumor necrosis factor-α.

**Table 6:** Salivary lactoferrin.

Authors (Year)	Group: No.	Saliva Type	Collection Method	Sodium Azide	Assessment Method	Main Findings
Antequera et al. (2024) [82]	EOAD: 28 LOAD: 25 YC: 59 OC: 45	Unstimulated whole saliva	Spit	Yes	ELISA	AD < Control EOAD > LOAD YC vs OC: No difference
Carro et al. (2017) [80]	AD: 80 Control: 91	Unstimulated whole saliva	Spit	Yes	ELISA	AD < Control
Gonzalez et al. (2020) [81]	AD: 25 Control: 118	Unstimulated whole saliva	Spit	Yes	ELISA	AD < Control
Gleerup et al. (2021) [83]	AD: 71 Control: 20	Unstimulated whole saliva	Spit	Unreported	ELISA	No difference
Zalewska et al. (2021) [21]	AD: 25 Control: 25	Stimulated whole saliva	Suction	Unreported	ELISA	AD < Control

Abbreviations: AD, Alzheimer’s Disease; ELISA, enzyme-linked immunosorbent assay; EOAD, Early-onset AD; LOAD, Late-onset AD; OC, Older controls; vs, versus; YC, Younger controls.

**Table 7:** Salivary melatonin.

Authors (Year)	Group: No.	Saliva Type	Collection Method	Assessment Method	Main Findings
Manni et al. (2019) [87]	AD: 21 Control: 17	Unreported	Unreported	ELISA	Dim light melatonin: AD < Control
Weissová et al. (2014) [88]	AD: 13 Control: 13	Unstimulated whole saliva	Spit	Radioimmunoassay	Daily melatonin: No difference

Abbreviations: AD, Alzheimer’s Disease; ELISA, enzyme-linked immunosorbent assay.

**Table 8:** Salivary cortisol.

Authors (Year)	Group: No.	Saliva Type	Collection Method	Assessment Method	Main Findings
Giubilei et al. (2001) [91]	AD: 18 Control: 18	Stimulated whole saliva	Polyester wool swab	Radioimmunoassay	AD > Control
James et al. (2019) [92]	AD: 65 Control: 69	Unstimulated whole saliva	Cotton	ELISA	No difference
Pena-Bautista et al. (2019) [93]	AD: 97 Control: 86	Unstimulated whole saliva	Spit	UPLC-MS/MS	No difference

Abbreviations: AD, Alzheimer’s Disease; ELISA, enzyme-linked immunosorbent assay; UPLC-MS/MS, ultra-performance liquid chromatography coupled to tandem mass spectrometry.

**Table 9:** Salivary oxidative stress markers.

Authors (Year)	Group: No.	Saliva Type	Collection Method	Assessment Method	Main Findings
Tvarijonaviciute et al. (2020) [45]	AD: 69 Control: 83	Unstimulated whole saliva	Spit	Colorimetric method	No difference: FRAP
Zalewska et al. (2021) [21]	AD: 25 Control: 25	Stimulated whole saliva	Suction	Colorimetric method	AD > Control: NO, TOS, OSI, Peroxynitrite
					AD < Control: GSH, UA
					No difference: TAC
				Spectrophotometric method	AD > Control: AGE, AOPP
					AD < Control: SOD, CAT, Px/GPx
				TBARS assay	AD > Control: MDA
				ELISA	AD > Control: Nitrotyrosine

Abbreviations: AD, Alzheimer’s Disease; AGE, advanced glycation end products; AOPP, advanced oxidation protein products; CAT, catalase; ELISA, enzyme-linked immunosorbent assay; FRAP, ferric reducing ability of plasma; GPx, glutathione peroxidase; GSH, glutathione; MDA, malondialdehyde; NO, nitric oxide; OSI, oxidative stress index; Px, peroxidase; SOD, superoxide dismutase; TAC, mean total antioxidant capacity; TBARS, thiobarbituric acid reactive substance; TOS, mean total oxidant status; UA, uric acid.

**Table 10:** The biomarkers of AD in salivary exosomes.

Authors (Year)	Group: No.	Saliva Type	Collection Method	Assessment Method	Main Findings
Rani et al. (2021) [99]	AD: 5 Control: 12	Unstimulated whole saliva	Spit	Western Blot	AD > Control: oligomeric Aβ, p-tau AD < Control: Aβ monomer
Ryu et al. (2023) [100]	AD: 27 Control: 13	Unreported	Oral swab	qPCR	AD > Control: miRNA-485-3p

Abbreviations: AD, Alzheimer’s Disease.

**Table 11:** The biomarkers of AD in salivary proteomics.

Authors (Year)	Group: No.	Saliva Type	Collection Method	Amylase Depletion	Assessment Method	Main Findings
Contini et al. (2021) [103]	AD: 35 Control: 35	Unstimulated whole saliva	Suction	Unreported	HPLC-ESI-IT-MS; Dot blotting	AD > Control: α-defensins, thymosin β4, cystatin B
Eldem et al. (2022) [101]	AD: 17 Control: 19	Unstimulated whole saliva	Unreported	Yes	LC-MS Western Blot	AD < Control: transthyretin
François et al. (2021) [102]	AD: 20 Control: 40	Unreported	Suction	Unreported	LC-MS	AD > Control: PKM, PGAM1, HSPA1A, MYL12B AD < Control: ALDH3

Abbreviations: AD, Alzheimer’s Disease; HPLC-ESI-IT-MS, high-performance liquid chromatography separation coupled to electrospray ion trap mass spectrometry; LC-MS, liquid chromatography mass spectrometry.

**Table 12:** The biomarkers of AD in salivary metabolomics.

Authors (Year)	Group: No.	Saliva Type	Collection Method	Refrain Smoking	Assessment Method	Main Findings
Huan et al. (2018) [108] and Sapkota et al. (2018) [110]	AD: 22 Control: 35	Unstimulated whole saliva	Spit	Unreported	LC-MS	AD > Control: methylguanosine, histidinyl-phenylalanine, choline-cytidine
Marksteiner et al. (2019) [106]	AD: 25 Control: 25	Unstimulated whole saliva	Spit	Yes	FIA-MS/MS	AD < Control: acyl-alkyl phosphatidylcholines
Yilmaz et al. (2017) [107]	AD: 9 Control: 12	Unstimulated whole saliva	Spit	Yes	NMR spectroscopy	AD > Control: propionate and acetone
Liang et al. (2015) [109]	AD: 256 Control: 218	Unstimulated whole saliva	Spit	Yes	FUPLC-MS	AD > Control: sphinganine-1-phosphate, ornithine, phenyllactic acid
François et al. (2021) [102]	AD: 20 Control: 40	Unreported	Unreported	Unreported	GC-MS	AD < Control: succinate, fumarate, L-lactate

Abbreviations: AD, Alzheimer’s Disease; FIA-MS/MS, flow injection analysis-tandem mass spectrometry; FUPLC-MS, faster ultra-high performance liquid chromatography-mass spectrometry; GC-MS, gas chromatograph-mass spectrometry; LC-MS, liquid chromatography-mass spectrometry; NMR, nuclear magnetic resonance.

**Table 13:** The characteristics of studies on the salivary microbiome in Alzheimer’s Disease.

Authors (Year)	Group: No.	Saliva Type	Collection Method	Antibiotics	Saliva Buffer	Dental Treatment	Teeth Number	Oral Health
Bathini et al. (2020) [114]	AD: 17 Control: 43	Unstimulated whole saliva	Spit	Unreported	Unreported	Unreported	Unreported	Unreported
Fu et al. (2022) [113]	AD: 20 Control: 20	Unstimulated whole saliva	Spit	3 months	TE	6 months	Unreported	No difference
Guo et al. (2021) [111]	AD: 26 Control: 26	Stimulated whole saliva	Spit	3 months	Saliva stabilizer	6 months	7	No difference
Liu et al. (2019) [112]	AD: 39 Control: 39	Unstimulated whole saliva	Spit	1 month	Unreported	2 months	Unreported	Unreported

Abbreviations: AD, Alzheimer’s disease; TE, Tris-EDTA.

**Table 14:** The biomarkers of the salivary microbiome in Alzheimer’s Disease.

Author (Year)	Assessment Method	Platform	Algorithm	Main Findings		
				α Diversity	β Diversity	Significant Bacteria
Bathini et al. (2020) [114]	V3-V4 16S rRNA sequencing	Illumina MiSeq	Unreported	Shannon: No difference	Unreported	AD < Control: *Filifactor villosus*
Fu et al. (2022) [113]	V3-V4 16S rRNA sequencing	Illumina MiSeq	out	Unreported	Difference	AD > Control: *Eubacterium infirmum*, *Prevotella buccae*, *Selenomonas artemidis*
Guo et al. (2021) [111]	16S rRNA full- length sequencing	PacBio platform	ASV	Unreported	No difference	AD > Control: *Veillonella parvula*
Liu et al. (2019) [112]	V3-V4 16S rRNA sequencing	Illumina Hiseq	OTU	Chao1: AD < Control Shannon: AD < Control	No difference	AD > Control: *Moraxella*, *Leptotrichia*, *Sphaerochaeta* AD < Control: *Rothia*

Abbreviations: AD, Alzheimer’s disease; ASV, Amplicon sequence variants; OTU, operational taxonomic unit; V3, third hypervariable region; V4, fourth hypervariable region; 16S rRNA,16S ribosomal RNA.

## Abbreviations

The following abbreviations are used in this manuscript:

Aβ	Amyloid-β peptide Aβ
AChE	Acetylcholinesterase
AchE-I	Acetylcholinesterase inhibitors
AD	Alzheimer’s disease
ADA	Adenosine deaminase
AGE	Advanced glycation end products
aMCI	Mild Cognitive Impairment due to AD
AOPP	Advanced oxidation protein products
APP	Amyloid precursor protein
ASV	Amplicon sequence variants (ASV)
CAT	Catalase
CC4	Complement C4;
CNS	Central nervous system
COX-2	Cyclooxygenase-2
CRP	C-reactive protein
CSF	Cerebrospinal fluid
CST-C	Cystatin-C
ELISA	Enzyme-linked immunosorbent assay
FIA-MS/MS	Flow injection analysis-tandem mass spectrometry
FRAP	Ferric reducing ability of plasma
FUPLC-MS	Faster ultra-high performance liquid chromatography-mass spectrometry
GC-MS	Gas chromatograph-mass spectrometry
GFAP	Glial fibrillary acidic protein
GPx	Glutathione peroxidase
GSH	Glutathione
Hp	Haptoglobin
HPLC-ESI-IT-MS	High-performance liquid chromatography separation coupled to electrospray ion trap mass spectrometry
IL-1	Interleukin-1
IL-1RN	Interleukin-1 receptor antagonist
IMA	Immunology multiplex assay
LC-MS/MS	Liquid-chromatography/mass spectroscopy
MDA	Malondialdehyde
MIP-4	Macrophage inflammatory protein-4
MMP-9	Matrix metalloproteinase 9
MMSE	Mini Mental State Examination
MNI	Magnetic nanoparticle immunoassay
NA	Not applicable
ncRNAs	non-coding RNAs
NfL	Neurofilament light chain
NIA-AA	National Institute on Aging Alzheimer’s Association
NINCDS–ADRDA	National Institute on Neurological Communicative Disorders and Stroke, and the Alzheimer’s Disease and Related Disorders Association
NMR	nuclear magnetic resonance
NO	Nitric oxide
OSI	Oxidative stress index
OTU	Operational taxonomic unit
PEDF	Pigment epithelium-derived factor
p-tau	Phosphorylated tau
Px	Peroxidase
SFN	Stratifin
Simoa	Single molecule array
SOD	Superoxide dismutase
16S rRNA	16S ribosomal ribosomal RNA
α-syn	α-synuclein
TAC	Mean total antioxidant capacity
TBARS	Thiobarbituric acid reactive substance
TNF	Tumor necrosis factor
TOS	Mean total oxidant status
t-tau	Total tau
UPLC-MS/MS	Ultra-performance liquid chromatography coupled to tandem mass spectrometry
UA	Uric acid
WHO	World Health Organization
V3	Third hypervariable region
V4	Fourth hypervariable region

## Appendix A

**Table A1:** The laboratory parameter data (mean ± standard deviation) and diagnostic performance of salivary Aβ.

Authors (Year)	AD	Control	*p*-Value	Sensitivity	Specificity	AUC
**Aβ42**						
Bermejo et al. (2010) [43] ^a^	7.67 ± 16.25	2.89 ± 4.96	**0.043**	0.16	0.93	0.547
Boschi et al. (2022) [38] ^a^	127.11 ± 33.44	66.11 ± 24.82	**<0.001**	0.84	0.68	/
Cui et al. (2022) [39]	/	/	**<0.05**	/	/	0.8483
Katsipis et al. (2021) [44] ^b^	10.43 ± 3.56	3.22 ± 1.13	**<0.0001**	/	/	/
Lee et al. (2017) [37] ^a,c^	59.07 ± 6.33	22.06 ± 0.4	**<0.001**	/	/	/
McGeer et al. (2020) [40] ^a^	51.70 ± 10.50	/	**<0.05**	/	/	/
Sabaei et al. (2023) [42] ^a,d^	104.3 ± 155.2	13.5 ± 21.5	**<0.001**	0.625	0.91	0.81
Sabbagh et al. (2018) [36] ^a^	51.7 ±1.6	21.1 ±0.3	**<0.05**	/	/	/
Tvarijonaviciute et al. (2020) [45] ^a^	3.15 ± 0.72	3.57 ± 0.93	**0.041**	/	/	/
**Aβ40**						
Bermejo et al. (2010) [43] ^a^	21.87 ± 5.7	20.82 ± 5.55	>0.05	/	/	/
Cui et al. (2022) [39]	/	/	>0.05	/	/	0.5311
Tvarijonaviciute et al. (2020) [45] ^a^	21.98 ± 16.94	19.97 ± 6.35	0.515	/	/	/

Note: ^a^ The measurement unit is pg/mL; ^b^ The measurement unit is pg/mg; ^c^ The data are shown as the mean ± standard error of the mean; ^d^ The data are shown as the median ± interquartile range.

**Table A2:** The laboratory parameter data (mean ± standard deviation) and diagnostic performance of salivary tau.

Authors (Year)	AD	Control	*p*-Value	Sensitivity	Specificity	AUC
**p-tau**						
Cui et al. (2022) [39]	/	/	>0.05	/	/	0.5831
Katsipis et al. (2021) [44] ^a^	33.87 ± 4.86	18.16 ± 5.67	**<0.0001**	/	/	/
Marksteiner et al. (2022) [48] ^a^	22.5 ± 3.6	9.7 ± 1.3	>0.05	/	/	/
Sabaei et al. (2023) [42] ^b,c^	9.2 ± 10.9	4.2 ± 6.1	**0.001**	0.917	0.638	0.78
Tvarijonaviciute et al. (2020) [45] ^b^	40.33 ± 42.95	42.5 ± 38.35	0.813	/	/	/
**t-tau**						
Cui et al. (2022) [39]	/	/	>0.05	/	/	0.505
Marksteiner et al. (2022) [48] ^a^	260 ± 53	577 ± 134	**<0.05**	/	/	/
Tvarijonaviciute et al. (2020) [45] ^b^	21.57 ± 22.11	21.15 ±16.58	0.923	/	/	/
**p-tau/t-tau**						
Cui et al. (2022) [39]	/	/	>0.05	/	/	0.6344
Marksteiner et al. (2022) [48] ^d^	41 ± 17	78 ± 17	>0.05	/	/	/
Pekeles et al. (2019) [54]	/	/	**<0.05**	0.73	0.5	/

Note: ^a^ The measurement unit is pg/mg; ^b^ The measurement unit is pg/mL; ^c^ The data are shown as the median+ interquartile range; ^d^ The presented data are the t-tau/p-tau ratio.

**Table A3:** The laboratory parameter data (mean ± standard deviation) and diagnostic performance of salivary biomarkers of neurodegeneration or neuronal injury.

Authors (Year)	Biomarker	AD	Control	Unit	*p*-Value	Sensitivity	Specificity	AUC
Gleerup et al. (2021) [58]	NfL	2.1 ± 1.6	2.3 ± 2.0	pg/mL	>0.05	/	/	/
Sabaei et al. (2023) [42]	α-syn	7.8 ± 6.6	12.5 ± 6.3	pg/mg	**<0.001**	0.667	0.682	0.71
Tvarijonaviciute et al. (2020) [45]	PEDF	31.41 ± −64.38	22.86 ± −49.21	pg/mL	0.52	/	/	/

Abbreviations: NfL, Neurofilament light chain; PEDF, Pigment epithelium-derived factor.

**Table A4:** The laboratory parameter data (mean ± standard deviation) and diagnostic performance of salivary acetylcholinesterase activity.

Authors (Year)	AD	Control	Unit	*p*-Value
Ahmadi et al. (2019) [64]	20.99 ± 10.99	13.08 ± 7.23	Unreported	**0.002**
Boston et al. (2008) [66]	0.039 ± 0.3	0.040 ± 0.044	a.u./50 μg	>0.05

**Table A5:** The laboratory parameter data (mean ± standard deviation) and diagnostic performance of salivary cytokines.

Authors (Year)	Biomarker	AD	Control	Unit	*p*-Value	Sensitivity	Specificity	AUC
Katsipis et al. (2021) [44]	IL-1	18.08± 29.03	281.2± 75.13	pg/mg	**<0.001**	/	/	/
	IL-6	14.58 ± 5.88	33.60 ± 3.56	pg/mg	**<0.001**	/	/	/
	TNF-α	2.44 ±1.69	10.01 ± 2.82	pg/mg	**<0.001**	/	/	/
	COX-2	81.06 ± 15.65 × 10^3^	50.28 ± 7.70 × 10^3^	pg/mg	**<0.001**	/	/	/
	Caspase-8	4.29 ± 1.53 × 10^3^	1.58 ± 0.77 × 10^3^	pg/mg	**<0.001**	/	/	/
	GFAP ^a^	3.56 ± 2.24	13.35 ± 3.03	ng/mg	**<0.0001**	0.75	1	/
	GFAP ^b^	4.57 ± 1.69	11.88 ± 2.42	ng/mg	**<0.0001**	0.85	0.75	/
Tvarijonaviciute et al. (2020) [45]	CRP	73.59 ± −64.1	57.38 ± −66.75	pg/mL	0.311	/	/	/
	α1 Antitrypsin	28.71 ± −108.62	17.89 ± −34.01	pg/mL	0.583	/	/	/
	MIP−4	0.49 ± −0.55	0.4 ± −0.57	pg/mL	0.496	/	/	/
	CC4	22.95 ± −17.66	15.37 ± −11.22	pg/mL	**0.048**	/	/	0.613
	ADA	7.61 ± −6.31	8.62 ± −11.05	IU/L	0.529	/	/	/
	Hp	2098.62 ± −1225.68	2252.91 ± −1510.63	ng/mL	0.532	/	/	/
Zalewska et al. (2021) [21]	IL-1β ^c^	88.47	70.58	ng/mg	**<0.0001**	0.84	0.84	0.8528

Abbreviations: ADA, adenosine deaminase; CC4, complement C4; COX-2, cyclooxygenase-2; CRP, C-reactive protein; GFAP, glial fibrillary acidic protein; Hp, haptoglobin; IL, interleukin; MIP-4, macrophage inflammatory protein-4; TNF, tumor necrosis factor. Note: ^a^ Biomarker was measured by enzyme-linked immunosorbent assay; ^b^ Biomarker was measured by Dot blotting; ^c^ The data represent the median.

**Table A6:** The laboratory parameter data (mean ± standard deviation) and diagnostic performance of salivary lactoferrin.

Authors (Year)	AD	Control	Unit	*p*-Value	Sensitivity	Specificity	AUC
Carro et al. (2017) [80]	4.78 ± 1.11	10.24 ± 1.96	μg/mL	**<0.001**	1	0.986	0.984
Gonzalez et al. (2020) [81]	67.2 ± 26.3	/	μg/mL	**<0.05**	0.8696	0.9167	0.95
Gleerup et al. (2021) [83]	26.9 ± 26.3	16.4 ± 6.6	μg/mL	>0.05	/	/	/
Zalewska et al. (2021) [21] ^a^	24.52	29.97	μg/mg	**0.0211**	0.64	0.64	0.6896

Note: ^a^ The data represent the median.

**Table A7:** The laboratory parameter data (mean ± standard deviation) and diagnostic performance of salivary cortisol.

Authors (Year)	AD	Control	Unit	*p*-Value
Giubilei et al. (2001) [91]	16.55 ± 12.38	10.31 ± 4.14	μg/dL	**<0.05**
James et al. (2019) [92] ^a^	0.82 ± 0.33	0.80 ± 0.31	/	0.761
Pena-Bautista et al. (2019) [93] ^b^	0.9	0.51	ng/mg	>0.05

Note: ^a^ Measurements are in nmol/L, data were log-transformed values; ^b^ The data represent the median.

**Table A8:** The laboratory parameter data (mean ± standard deviation) and diagnostic performance of salivary oxidative stress markers.

Authors (Year)	Biomarker	AD	Control	*p*-Value	Sensitivity	Specificity	AUC
Tvarijonaviciute et al. (2020) [45]	FRAP	0.94 ± −0.55	1.06 ± −0.74	0.274	/	/	/
Zalewska et al. (2021) [21]	SOD	/	/	**0.007**	0.6957	0.68	0.7774
	CAT	/	/	**<0.0001**	0.8261	0.84	0.9183
	GPx	/	/	**0.0037**	0.7391	0.72	0.7409
	UA	/	/	0.38955	0.5217	0.52	0.5739
	GSH	/	/	**0.03122**	0.7273	0.72	0.6836
	TAC	/	/	0.838	0.5217	0.52	0.5183
	TOS	/	/	**<0.0001**	0.913	0.92	0.92
	OSI	/	/	**<0.0001**	0.9	0.92	0.936
	AGE	/	/	**<0.0001**	0.8696	0.88	0.9357
	AOPP	/	/	**0.0285**	0.56	0.56	0.68
	MDA	/	/	**0.0297**	0.6667	0.68	0.6876
	NO	/	/	**0.0371**	0.56	0.56	0.672
	Peroxynitrite	/	/	**0.0001**	0.6364	0.7917	0.8163
	Nitrotyrosine	/	/	**0.0175**	0.6364	0.64	0.7018

Abbreviations: AGE, advanced glycation end products; AOPP, advanced oxidation protein products; CAT, catalase; FRAP, ferric reducing ability of plasma; GPx, glutathione peroxidase; GSH, glutathione; MDA, malondialdehyde; NO, nitric oxide; OSI, oxidative stress index; SOD, superoxide dismutase; TAC, mean total antioxidant capacity; TOS, mean total oxidant status; UA, uric acid.

**Table A9:** The laboratory parameter data (mean ± standard deviation) and diagnostic performance of miRNA 485-3p concentrations in salivary exosomes.

Authors (Year)	AD	Control	Unit	*p*-Value	Sensitivity	Specificity	AUC
Ryu et al. (2023) [100]	0.0483 ± 0.0278	0.0205 ± 0.0082	Pg	**<0.05**	0.7407	0.9231	0.775

**Table A10:** The laboratory parameter data (mean ± standard deviation) and diagnostic performance of biomarkers of AD in salivary proteomics.

Authors (Year)	Biomarker	AD	Control	*p*-Value	Sensitivity	Specificity	AUC
Contini et al. (2021) [103] ^a^	α-defensins	3.9 ± 4.0 × 10^5^	1.2 ± 1.5 × 10^5^	**0.0005**	/	/	/
	thymosin β4	0.7 ± 0.8 × 10^5^	0.2 ± 0.4 × 10^5^	**0.0005**	/	/	/
	cystatin B	1.7 ± 1.9 × 10^5^	0.6 ± 0.6 × 10^5^	**0.002**	/	/	/
Eldem et al. (2022) [101] ^b,c^	TTR	0.519 ± 0.107	0.99 ± 0.149	**<0.05**	/	/	/

Note: ^a^ The data represent the extracted Ion Current peak area values; ^b^ The data represent the optical density; ^c^ The data represent the mean ± standard error of the mean.

**Table A11:** The fold change and diagnostic performance of biomarkers of AD in salivary metabolomics.

Authors (Year)	Biomarker	AD/Control Fold Change	*p*-Value	Sensitivity	Specificity	AUC
Huan et al. (2018) [108] and Sapkota et al. (2018) [110]	Methylguanosine	4.28	**<0.05**	0.9852	0.9655	0.997
	Histidinyl- Phenylalanine	5.06	**<0.05**			
	Choline-cytidine	4.39	**<0.05**			
Liang et al. (2015) [109]	sphinganine-1- phosphate	12.11	**<0.05**	0.994	0.982	0.998
	ornithine	3.94	**<0.05**	0.819	0.907	0.927
	phenyllactic	3.44	**<0.05**	0.795	0.843	0.831
Yilmaz et al. (2017) [107]	propionate and acetone	/	**<0.05**	0.9	0.944	0.897

**Table A12:** The laboratory parameter data (mean ± standard error of the mean) of biomarkers in Salivary metabolomics (acyl-alkyl phosphatidylcholines).

Authors (Year)	Biomarker	AD	Control	Unit	*p*-Value
Marksteiner et al. (2019) [106]	PCae C34:(1 + 2)	358 ± 80	985 ± 233	μM	**0.008**
	PCae C36:(1 + 2 + 3)	224 ± 34	593 ± 108	μM	**0.0011**
	PCae C38:(1 + 3)	57 ± 10	135 ± 27	μM	**0.009**
	PCae C40:(2 + 3)	53 ± 11	128 ± 27	μM	**0.011**
